# Transcatheter tricuspid valve replacement in arrhythmogenic right ventricular cardiomyopathy after prior cardioband annuloplasty: insights from the first-in-man case report

**DOI:** 10.1093/ehjcr/ytag424

**Published:** 2026-06-09

**Authors:** Jonas Michael Bodanowitz, Plamen Kochev, Benjamin Storek, Antonia Ourani, Hüseyin Ince

**Affiliations:** Department of Cardiology, Universitätsmedizin Rostock, Schillingallee 36, Rostock 18057, Germany; Department of Cardiology, Universitätsmedizin Rostock, Schillingallee 36, Rostock 18057, Germany; Department of Geriatrics, Vivantes Ida-Wolff-Krankenhaus, Zadekstraße 46, Berlin 12351, Germany; Department of Cardiology, Universitätsmedizin Rostock, Schillingallee 36, Rostock 18057, Germany; Department of Cardiology, Vivantes Klinikum Am Urban, Dieffenbachstraße 1, Berlin 10967, Germany; Department of Cardiology, Vivantes Klinikum Neuköln, Rudower Str. 48, Berlin 12351, Germany; Department of Cardiology, Universitätsmedizin Rostock, Schillingallee 36, Rostock 18057, Germany; Department of Cardiology, Vivantes Klinikum Am Urban, Dieffenbachstraße 1, Berlin 10967, Germany; Department of Cardiology, Vivantes Klinikum Neuköln, Rudower Str. 48, Berlin 12351, Germany

**Keywords:** Arrhythmogenic right ventricular cardiomyopathy (ARVC), Cardioband annuloplasty, Severe tricuspid regurgitation, Transcatheter tricuspid valve replacement (TTVR), Right heart failure, Case report

## Abstract

**Background:**

Severe tricuspid regurgitation (TR) in patients with arrhythmogenic right ventricular cardiomyopathy (ARVC) represents a particularly challenging clinical scenario due to advanced right ventricular dysfunction, complex device interactions, and high procedural risk. Evidence regarding transcatheter tricuspid valve replacement (TTVR) in ARVC, especially following prior transcatheter annuloplasty procedures such as Cardioband, remains scarce.

**Case summary:**

We present the first in-man case report of a successful transfemoral TTVR using the EVOQUE™ system in a 69-year-old patient with ARVC, severe TR, heart failure symptoms corresponding to New York Heart Association (NYHA) class IV, prior Cardioband annuloplasty, and an implantable cardioverter-defibrillator (ICD). The patient presented with advanced right heart failure requiring intensive pre-procedural stabilization. TTVR with a 56-mm EVOQUE™ valve resulted in an immediate reduction of TR from grade V to grade 0. The post-procedural course was complicated by severe right heart failure, respiratory insufficiency requiring prolonged mechanical ventilation, ventricular arrhythmias associated with subtherapeutic amiodarone levels, and acute kidney injury requiring temporary renal replacement therapy. Following multidisciplinary intensive care management, the patient stabilized with marked clinical improvement and sustained valve function.

**Discussion:**

This case highlights the feasibility of EVOQUE™ TTVR in patients with prior annuloplasty devices and advanced ARVC. It demonstrates the substantial risk of post-procedural right heart failure and complex device interactions, emphasizing that procedural success does not preclude a complicated clinical course. Careful patient selection, procedural planning, and intensive post-procedural management are essential in this high-risk population.

Learning points

- Transcatheter tricuspid valve replacement with EVOQUE™ is feasible in patients with ARVC and prior annuloplasty devices such as Cardioband.
- Patients with advanced ARVC and prior annuloplasty devices, such as Cardioband, undergoing TTVR are at high risk of acute post-procedural right heart failure.
- Complex interactions between prosthetic valves, annuloplasty devices, and ICD leads require individualized and multidisciplinary treatment strategies.

## Introduction

Arrhythmogenic right ventricular cardiomyopathy (ARVC) is characterized by progressive fibrofatty replacement of the myocardium, leading to right ventricular (RV) dilation, dysfunction, and ventricular arrhythmias.^1–3^ Functional tricuspid regurgitation (TR) frequently develops in advanced stages due to annular dilation and leaflet tethering and is associated with adverse clinical outcomes.^4^

Transcatheter repair techniques, including edge-to-edge repair and annuloplasty systems such as Cardioband, have expanded treatment options for functional TR. However, their efficacy is limited in patients with advanced disease, characterized by severe annular dilation and leaflet tethering.^5^ In such cases, transcatheter tricuspid valve replacement (TTVR) has emerged as a promising alternative, with recent studies demonstrating high procedural success rates and symptomatic improvement.^6,7^

Our initial report demonstrated the feasibility of TTVR in a patient with ARVC and severe TR.^8^ However, evidence in more complex scenarios, particularly in the presence of prior annuloplasty devices and severe right ventricular dysfunction, remains extremely limited.

We report the first case of successful EVOQUE™ TTVR in a patient with ARVC after prior Cardioband annuloplasty, highlighting procedural feasibility and the challenges of managing advanced right heart failure in this high-risk population.

## Summary figure

**Figure:**
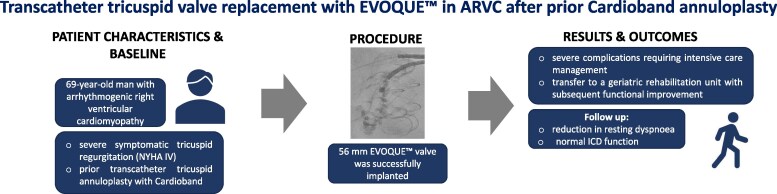


## Case presentation

**Figure 1:**
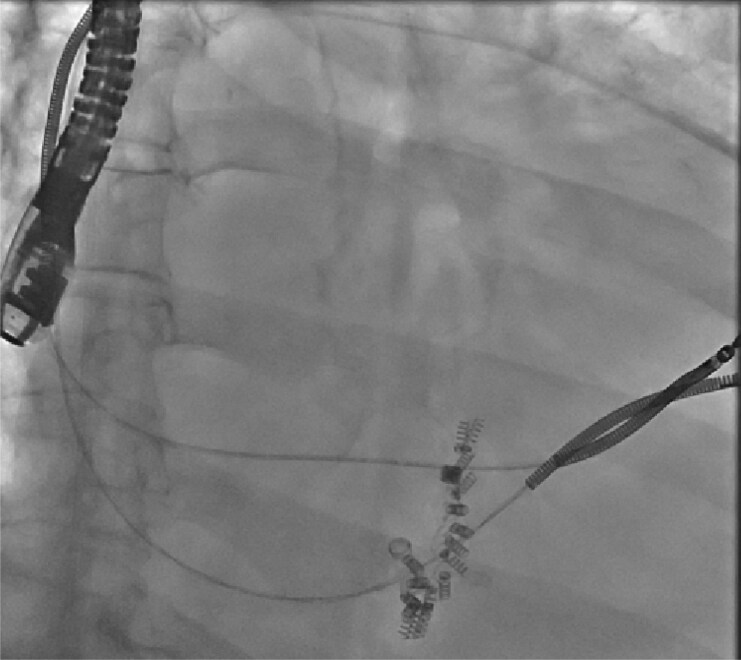
Intraprocedural fluoroscopic image during EVOQUE™ tricuspid valve implantation showing prior cardioband annuloplasty and ICD leads.

A 69-year-old man with advanced arrhythmogenic right ventricular cardiomyopathy (diagnosed in 2008) was admitted for elective evaluation of transcatheter tricuspid valve replacement due to severe symptomatic tricuspid regurgitation (NYHA IV). His medical history included ICD implantation for recurrent ventricular tachycardia in 2008, prior generator replacements, and transcatheter tricuspid annuloplasty with Cardioband in 2022 (*Figure 1*). The prior Cardioband annuloplasty had been performed at an external institution; therefore, the original procedural indication and decision-making process were not fully available to our team. Coronary angiography had previously demonstrated single-vessel coronary artery disease without evidence of haemodynamically significant stenosis. Pre-procedural spirometry showed no evidence of obstructive ventilatory impairment. Additional comorbidities included chronic kidney disease, type 2 diabetes mellitus, arterial hypertension, prior ischaemic stroke with residual deficits, and suspected chronic obstructive pulmonary disease.

**Figure 2:**
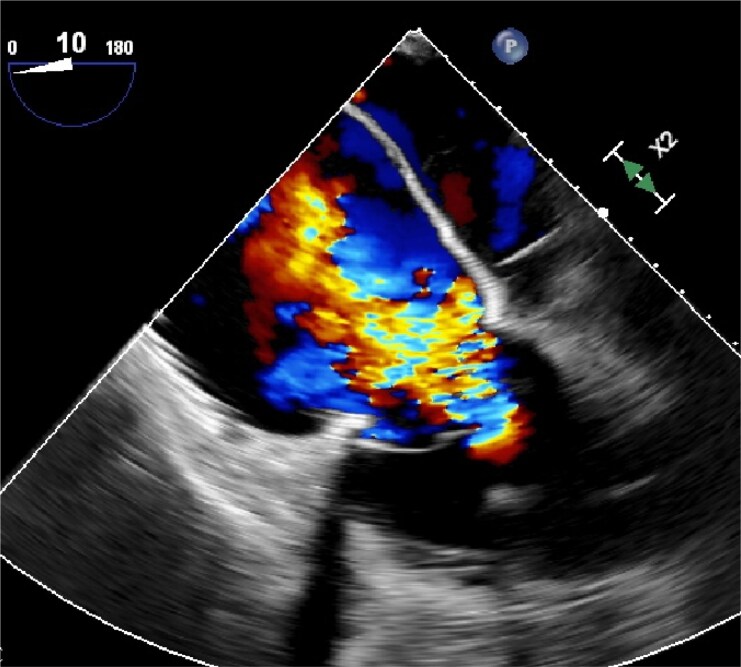
Transoesophageal echocardiography demonstrating severe tricuspid regurgitation prior to EVOQUE™ implantation.

At presentation, the patient showed signs of advanced right heart failure with marked peripheral oedema, progressive weight gain, and severely reduced exercise capacity. Electrocardiography at admission showed atrial fibrillation with a controlled ventricular response. Echocardiography demonstrated severe TR (grade V) with massive right atrial and ventricular dilation and an RV end-diastolic diameter (RVEDD) of 60 mm and impaired RV function (TAPSE of 9 mm) (*Figure 2*). Despite intensified intravenous diuretic therapy including sequential nephron blockade, clinical deterioration persisted. Following Heart Team discussion, transfemoral TTVR using the EVOQUE™ system was recommended due to prohibitive surgical risk and failure of prior annuloplasty therapy.

**Figure 3:**
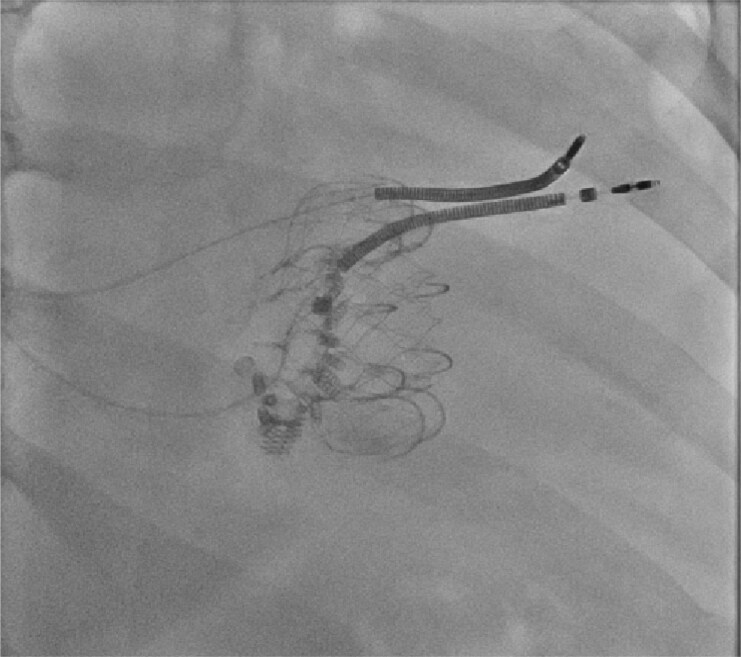
Intraprocedural fluoroscopic image during EVOQUE™ tricuspid valve implantation.

**Figure 4:**
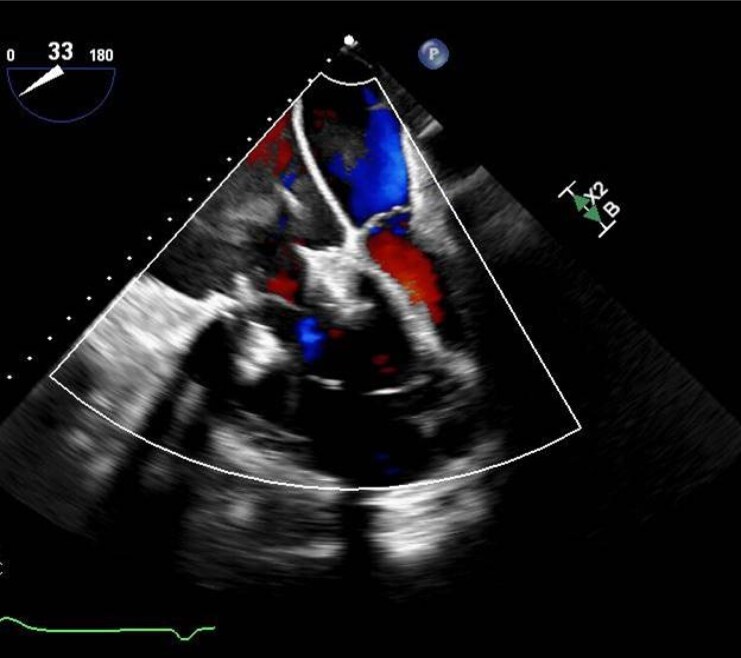
Transoesophageal echocardiography after EVOQUE™ implantation showing no evidence of tricuspid regurgitation.

The procedure was performed under general anaesthesia via right femoral venous access using a 28-French delivery system. A 56-mm EVOQUE™ valve was successfully implanted under fluoroscopic and transoesophageal echocardiographic guidance. Post-deployment imaging demonstrated excellent positioning with immediate reduction of TR from grade V to grade 0 (*Figures 3* and *4*).

Post-procedurally, the patient developed significant haemodynamic instability requiring prolonged catecholamine support with dobutamine and norepinephrine. During the early post-interventional period, an increased incidence of nosocomial viral pulmonary infections occurred in the hospital, followed by bacterial superinfection in our patient, resulting in progressive respiratory failure due to pulmonary oedema and pneumonia. Consequently, invasive mechanical ventilation was required for two weeks, alongside escalation of anti-infective therapy, which ultimately led to clinical improvement. The total intensive care unit stay was approximately three weeks. The clinical course was further complicated by recurrent ventricular tachycardia episodes in the setting of hypokalaemia and subtherapeutic amiodarone levels, requiring intravenous loading and electrical cardioversion. Device interrogation revealed impaired sensing of the pre-existing ICD lead, which had already been present before the intervention. Lead extraction before TTVR was discussed as a potential strategy; however, the patient declined lead extraction due to the associated procedural risks in the setting of advanced ARVC and severe right ventricular dysfunction, including the potential risk of leaflet injury caused by adherence of the valve leaflets to the pacemaker lead, which could render subsequent valve implantation challenging or even impossible. No new rhythm disturbances attributable to the EVOQUE™ implantation were observed. Due to acute kidney injury (AKIN stage III), temporary continuous venovenous haemofiltration was initiated. Gastrointestinal dysmotility required placement of a triple-lumen feeding tubefeeding tube. During recovery, the patient developed delirium associated with a urinary tract infection, which resolved under targeted therapy.

With gradual haemodynamic stabilization and intensive multidisciplinary management, the patient improved clinically. A cumulative negative fluid balance of approximately 15 kg was achieved. Anticoagulation with apixaban was initiated following valve implantation, consistent with current clinical practice.

At discharge, the patient was haemodynamically stable, did not require supplemental oxygen, and showed marked clinical improvement. The patient was subsequently transferred to a geriatric rehabilitation unit for further supportive care. During rehabilitation, a rapid improvement in functional status and mobility was observed, with a particularly pronounced and clinically relevant resolution of the previously present resting dyspnoea.

At 6-week follow-up, echocardiography demonstrated the prosthetic valve in a stable position with favourable haemodynamic parameters. Device interrogation revealed normal ICD function with stable sensing and pacing parameters. In the event of future ICD dysfunction, implantation of a subcutaneous ICD (S-ICD) is considered a potential therapeutic option. The marked improvement in resting dyspnoea represented a major clinical benefit, enabling the patient to speak without respiratory discomfort and to participate in conversations in daily life, which had not been possible before the intervention and therefore constituted a significant gain in quality of life. Moreover, improvement in renal function despite longstanding chronic kidney disease, and controlled weight reduction of 15 kg during hospitalization, achieved through tailored diuretic therapy, strongly underscores that treatment of tricuspid regurgitation may provide substantial clinical benefit even in the setting of very advanced right heart failure in ARVC patients.

## Discussion

This case highlights several important aspects of transcatheter tricuspid valve replacement (TTVR) in highly complex patients with advanced right heart disease.

First, it demonstrates the feasibility of EVOQUE™ implantation in the presence of prior annuloplasty devices such as Cardioband in patients with ARVC. Annuloplasty systems aim to reduce annular dimensions and improve leaflet coaptation; however, their efficacy is limited in advanced disease stages characterized by severe annular dilation and leaflet tethering.^5^ In such anatomically complex settings, valve replacement strategies may represent a more definitive therapeutic option. While our initial report has demonstrated the feasibility of TTVR in a patient with ARVC, evidence following prior annuloplasty procedures remains scarce.^8^ The feasibility of TTVR in patients with prior failed edge-to-edge repair has already been described and discussed in several case reports.^9,10^ This case highly expands the applicability of TTVR in ARVC to a particularly challenging subgroup. To our knowledge, no publications or clinical experience exist regarding this specific constellation in ARVC patients with prior annuloplasty, making this case unique

Second, patients with advanced ARVC represent a high-risk population due to severely impaired right ventricular (RV) reserve. The abrupt elimination of severe TR may lead to acute afterload mismatch, resulting in haemodynamic instability and right heart failure.^11,12^ In contrast to previously reported cases with relatively stable post-procedural courses, our patient developed severe right heart failure requiring prolonged intensive care management, including vasopressor support, invasive mechanical ventilation, and renal replacement therapy. This course was further complicated by an intercurrent infectious process, which significantly aggravated right heart failure and contributed to prolonged haemodynamic instability. This highlights the importance of anticipating post-procedural haemodynamic deterioration and highlights that successful valve implantation does not preclude a complicated clinical course in patients with advanced RV disease. This emphasizes that patient selection and peri-procedural management are critical determinants of outcomes. After successful anti-infective therapy, catecholamine support was rapidly weaned. We assume that elimination of TR may have contributed to a faster haemodynamic stabilization and potentially prevented an even more prolonged hospital stay.

Third, this case illustrates the complexity of device–device interactions in contemporary structural heart interventions. The coexistence of ICD leads, prior annuloplasty systems, and a transcatheter valve requires meticulous procedural planning and careful follow-up. Although no immediate device-related complications occurred during implantation, pre-existing ICD lead dysfunction became clinically relevant during the post-procedural course. Similar challenges have been described in patients undergoing transcatheter tricuspid interventions, emphasizing the need for individualized management strategies and close interdisciplinary collaboration.^13^ Device interrogation at 6-week follow-up revealed normal ICD function with stable sensing and pacing parameters. In the event of future ICD dysfunction, implantation of a subcutaneous ICD (S-ICD) is considered a potential therapeutic option.

Fourth, ARVC-specific procedural risks must be considered. Progressive fibrofatty myocardial replacement may increase the risk of RV injury during wire manipulation and device deployment, while instrumentation of the right ventricle carries a potential pro-arrhythmic risk. Although no procedure-related ventricular arrhythmias occurred in this case, the patient developed recurrent ventricular tachycardia episodes in the post-procedural phase, primarily driven by metabolic disturbances and subtherapeutic antiarrhythmic drug levels. This highlights the importance of meticulous rhythm monitoring and optimized medical therapy in the peri-procedural period. Importantly, this case also demonstrates that right-sided transcatheter valve interventions can be successfully performed even in highly selected high-risk ARVC patients with prior right-sided annuloplasty systems such as Cardioband, when undertaken within a carefully structured multidisciplinary and peri-procedural management framework.

Finally, this case underscores the importance of multidisciplinary management in patients undergoing TTVR. Despite severe complications, including respiratory failure, infection, arrhythmias, and renal dysfunction, a favourable outcome was achieved through coordinated intensive care, heart failure therapy, and rhythm management. Uncertainties remain regarding long-term durability, the impact on RV remodelling, and the interaction between transcatheter valves and pre-existing devices. Further data from registries and longer follow-up are required to better define the role of TTVR in advanced ARVC, particularly regarding its potential to provide sustained symptomatic and haemodynamic benefit.

## Conclusion

TTVR with the EVOQUE™ system is feasible in patients with ARVC and prior annuloplasty devices such as Cardioband. However, these patients are at high risk for post-procedural complications, particularly right heart failure, and require intensive multidisciplinary care. This case adds to the current evidence for TTVR in complex structural and device-related scenarios. Importantly, in our patient, the intervention ultimately provided a meaningful clinical benefit despite the high procedural risk, with a marked improvement in pre-existing resting dyspnoea and a consequent significant enhancement in quality of life.

## Data Availability

The data underlying this article will be shared on reasonable request to the corresponding author.

## Lead author biography

Hüseyin Ince is a Professor of Cardiology (since 2008). In October 2013, he served as Director of the Department of Cardiology at Vivantes Klinikum Am Urban and Klinikum im Friedrichshain in Berlin. Since April 2017, he serves as Director of the Clinic of Cardiology at the University of Rostock. In addition, he serves as Director of the Department of Cardiology at Vivantes Klinikum Am Urban and, since 2022, also at Vivantes Klinikum Neukölln in Berlin.

## Notes

**Consent:** The authors confirm that written consent for submission and publication of this case report including images and associated text has been received from the patient in line with the Committee on Publication Ethics (COPE) guidelines.
